# Supplementary material for: Causal Relationship between Aging and Anorexia Nervosa: A White-Matter-Microstructure-Mediated Mendelian Randomization Analysis
Source: Biomedicines. 2024 Aug 16;12(8):1874. doi: 10.3390/biomedicines12081874 (PMC11351342; doi:10.3390/biomedicines12081874)
Supplement: Supplementary file 1 [file biomedicines-12-01874-s001.zip › Supplementary Figure/Figure S9/Leaveoneout plot of MR analysis for ubm-b-1624 to AN.pdf]

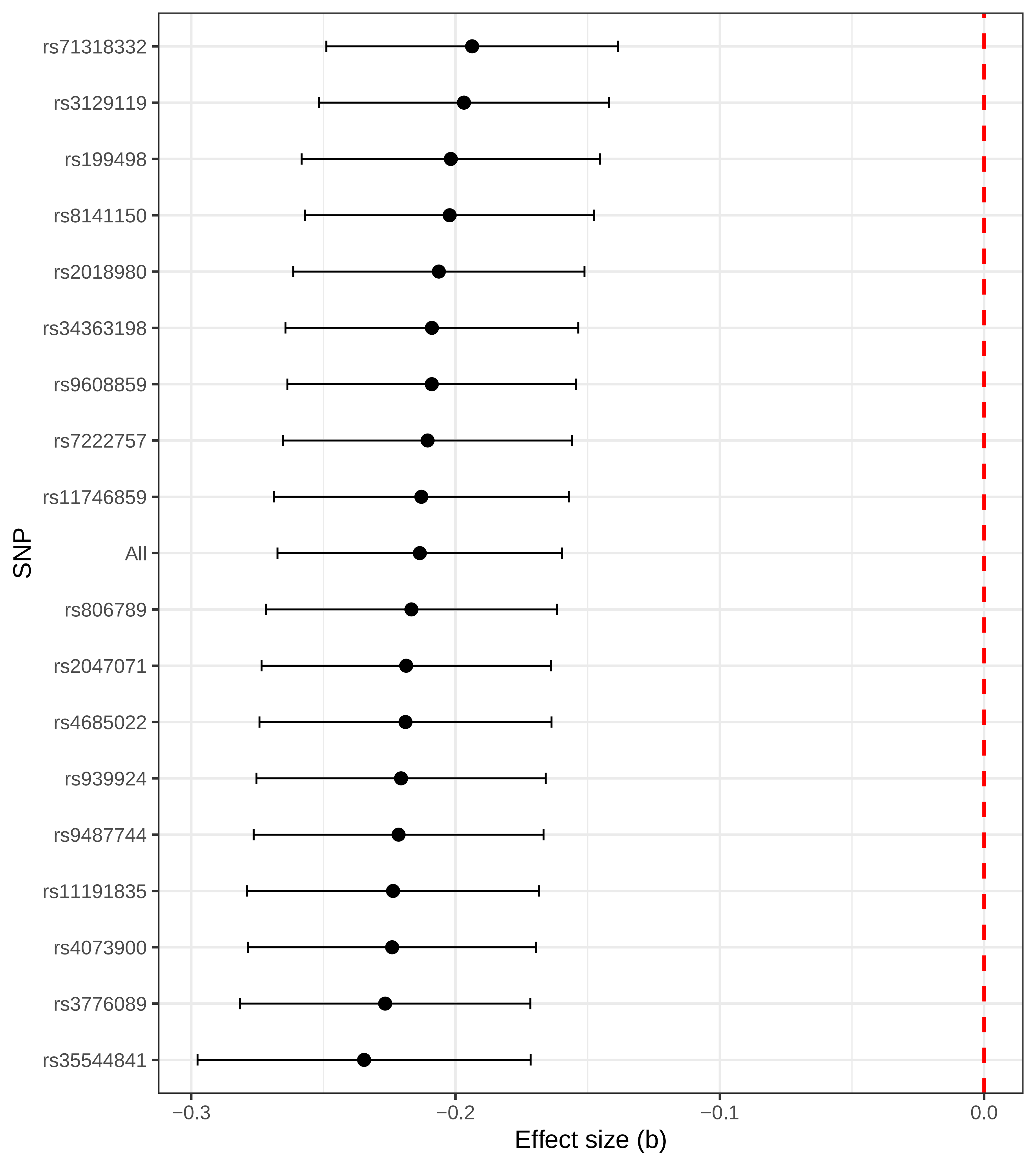

rs71318332
rs3129119
rs199498
rs8141150
rs2018980
rs34363198
rs9608859
rs7222757
rs11746859
All
rs806789
rs2047071
rs4685022
rs939924
rs9487744
rs11191835
rs4073900
rs3776089
rs35544841
SNP
−0.3
−0.2
−0.1
0.0
Effect size (b)
